# Zebrabase: An Intuitive Tracking Solution for Aquatic Model Organisms

**DOI:** 10.1089/zeb.2018.1609

**Published:** 2018-11-29

**Authors:** Jana Oltova, Jindrich Jindrich, Ctibor Skuta, Ondrej Svoboda, Olga Machonova, Petr Bartunek

**Affiliations:** ^1^ Department of Cell Differentiation, Institute of Molecular Genetics AS CR v.v.i., Prague, Czech Republic.; ^2^ CZ-OPENSCREEN, Institute of Molecular Genetics AS CR v.v.i., Prague, Czech Republic.; ^3^ Department of Organic Chemistry, Faculty of Science, Charles University, Prague, Czech Republic.

**Keywords:** zebrafish, tracking, husbandry, facility database, Django, Python

## Abstract

Small fish species, such as zebrafish and medaka, are increasingly gaining popularity in basic research and disease modeling as a useful alternative to rodent model organisms. However, the tracking options for fish within a facility are rather limited. In this study, we present an aquatic species tracking database, Zebrabase, developed in our zebrafish research and breeding facility that represents a practical and scalable solution and an intuitive platform for scientists, fish managers, and caretakers, in both small and large facilities. Zebrabase is a scalable, cross-platform fish tracking database developed especially for fish research facilities. Nevertheless, this platform can be easily adapted for a wide variety of aquatic model organisms housed in tanks. It provides sophisticated tracking, reporting, and management functions that help keep animal-related records well organized, including a QR code functionality for tank labeling. The implementation of various user roles ensures a functional hierarchy and customized access to specific functions and data. In addition, Zebrabase makes it easy to personalize rooms and racks, and its advanced statistics and reporting options make it an excellent tool for creating periodic reports of animal usage and productivity. Communication between the facility and the researchers can be streamlined by the database functions. Finally, Zebrabase also features an interactive breeding history and a smart interface with advanced visualizations and intuitive color coding that accelerate the processes.

## Introduction

Zebrafish is a popular vertebrate model organism widely used in scientific research and a highly prized disease model. Thanks to its high fecundity, optical transparency, and rapid development, zebrafish is highly attractive model for live imaging and developmental studies. Especially the recent, rapid progress in the field of genome editing has prompted us to develop a database system that would facilitate fish tracking of the F0 and F1 generations of newly created, genetically modified lines, which is both highly laborious and space demanding and requires a high level of organization in the facility. Furthermore, proper outcrossing is required in zebrafish to prevent the inbreeding depression of fish stocks,^1^ which is particularly prone to mix-ups.

Despite the increasing use of the zebrafish model in research, the currently available tracking options suitable for aquatic animals housed in tanks remain rather limited, and scientists often depend on outdated software solutions or simple spreadsheets. An explanation for this limitation could be that the requirements for aquatic organism tracking significantly differ from the rodent tracking, wherein systems such as PyRat (www.scionics.com/pyrat.html) or open-source JAX Colony Management System^2^ (http://colonymanagement.jax.org) have long been established as a standard. The main specificity of aquatic animal tracking is group tracking, as opposed to tracking individual animals. One of the reasons for group tracking is that, up to date, there is no cost-effective system designed to distinguish single fish individuals in a tank. Technologies such as subdermal chipping are developing fast, but the technology is not yet sufficiently miniaturized to enable its routine use in tracking small fish species, although some significant advances have been reported using the radio frequency identification microtags.^3^ In addition, due to the social requirements and bulk mating, zebrafish should be kept within a specific density range in the with both males and females.^4,5^ Keeping individual fish in solitude adversely affects their development and health^6^ and, moreover, is very space inefficient.

A handful of solutions, either commercial (e.g., www.daniodata.com, www.scionics.com/pyrat_aquatic.html) or open-source^7–9^ (http://zebase.bio.purdue.edu, http://aqacs.uoregon.edu/zf/files, https://zebrafish.jimdo.com) are currently available. However, none of them is prevalent in the zebrafish community most likely because each of them is tailored to a slightly different set of requirements of the facilities. Two main drawbacks of the solutions currently available in the market are the price and dedication to a single platform, which can both be perceived as a limiting factor, especially for small facilities with a limited budget. The sustainability and implementation of new functions have been the bottleneck of some databases developed in the recent years, and this aspect should indeed be carefully considered because switching from one system to another can be rather tedious, especially when aiming to save the tracking history. Finally, as reporting requirements from local authorities are becoming increasingly rigorous in many countries, another feature missing in some of the existing solutions is a detailed fish usage and health reporting capability. In this study, we present Zebrabase, a novel tracking database for zebrafish housing facilities.

## Results

### General concept

Zebrabase is a web-based, cross-platform hosted zebrafish tracking solution supporting both desktop and mobile use on Mac, Linux, Windows, Android, and iOS. The database is accessible via an optimized user interface, which serves both for browsing fish stock records and creating new ones via the action menu. Moreover, Zebrabase has a built-in QR code generator and QR code reader functionality that provide instant access to all fish stock information and related actions on a mobile device.

### Basic operation principles

Zebrabase enables users to track animals from birth to death and to store detailed records of all relevant events. Every action performed by the user creates a record in the database and can be later used for reporting purposes. Zebrabase is optimized for touch devices and supports the use of QR codes for tank labeling to efficiently track animals.

### Fish characterization and grouping

The most important purpose of Zebrabase is the time-efficient and comprehensive tracking of fish stocks. Therefore, all animals in a facility are divided into fishlines specified by the database administrator, who defines their genotypes, the date of creation or import and other optional information (https://public.zebrabase.org/fishline/create). Fish corresponding to a single fishline are further organized into groups of siblings, stocks, sharing the same date of birth, and parents. Because the number of fish in some stocks might exceed the capacity of a single tank, we introduced another level of grouping animals, substocks, which refer to a defined stock subset in a single tank. Thus, substock is the basic unit tracked in the database consisting of a variable number of fish individuals.

Genotypes consist of a modification category (transgene, natural mutant, engineered mutant, or custom) and either of a driver, triggered gene, and an optional allele for transgenic genotypes or of an affected gene and allele/mutation for mutant genotypes. The Zebrabase name generation rules are based on the common ZFIN nomenclature (https://wiki.zfin.org/display/general/ZFIN+Zebrafish+Nomenclature+Guidelines). An alias can also be specified and used as a trivial name for substocks with very long generated names (e.g., in the case of multiple genotypes) or a suffix can be appended to the generated name.

Zebrabase enables users to store attachments for each fishline to effectively organize genotyping protocols, publications, and all visual content, for example, phenotypes of mutant lines or transgene expression patterns used as a sorting reference for reporter lines. To achieve an additional degree of organization of the fishlines, a responsible user, and a workgroup can be specified.

### Spatial and temporal tracking

**FIG. 1.**
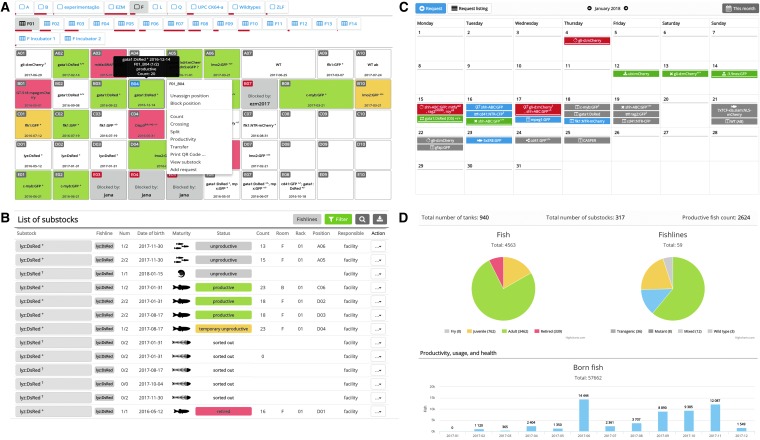
Zebrabase user interface. **(A)** Facility view is a mobile-optimized visual facility representation that enables users to browse all positions within the facility and to perform all actions. *Rows* are identified by *letters* and *horizontal* positions by *numbers*. Combined with the rack ID, each position is uniquely defined within the whole facility. Upon click, the action menu appears and enables users to perform all actions available for a particular substock. **(B)** Fish list is a list of all records in the facility, regardless of whether they have a specific position in the facility or not. Records can be intuitively filtered, according to various parameters, to create a subset of fish of our interest. By default, only active (alive) fish are shown in the list, but that can be changed by adjusting the filter. Substocks are color-coded in the following manner: *green*—productive, *dark gray*—blocked, *light gray*—unproductive, *red*—retired, and *yellow*—temporary productivity issues. **(C)** Representation of various requests and self-requests in the calendar. Various icons are used for various types of requests (fish, setup fish, crossing, terminate, genotyping, mobilize fish, transfer, import, export). The requests are color-coded in the following manner: *gray*—new requests, *blue*—accepted requests, *green*—performed requests, and *red*—declined requests. **(D)** Zebrabase statistics enables users to store, visualize and export data for reporting purposes. The overview of facility statistics is presented as pie charts. *Left*—overview of the age distribution of the fish in the facility. *Right*—overview of the fishlines in the facility. *Bottom*—the bar charts are provided and plotted monthly for fish born, deceased, euthanized, and used for experiments in the facility (images not shown). Figure 1 can be viewed in greater detail online at www.liebertpub.com/zeb

We have developed a system for tracking animals in laboratory, which uses the positional widget termed facility (Fig. 1A), a platform for user-defined rooms, racks, and tanks. In facility, the productivity of substocks is visualized by consistent color coding to provide a quick status reference for users. We have also optimized the QR code functionality to efficiently record the action history of each substock via any mobile device, as an alternative to the manual entering from a desktop computer. In addition, a list view of all substocks, regardless of their position in the facility, including deceased or terminated substocks, can be found under the fish tab (Fig. 1B).

An interactive breeding history diagram is shown at the substock record page (https://public.zebrabase.org/substock/1673), which provides links to all substocks in the whole pedigree. This diagram also shows the dates of crosses and splits and, when clicked, leads to the action detail page. Breeding history enables to efficiently track animals that may have shared the same housing or breeding tank (e.g., during outcrosses) when requiring to adopt a cautious approach upon disease outbreak or potential fish mix-ups, among others.

Zebrabase also features a number of actions—crossing, split, transfer, count, productivity, genotyping, add request, and print QR code. These actions can be fully tracked, browsed, or filtered at any time, when requiring to trace a specific event. Action history is also listed on the substock record page to provide a full overview of all actions performed with a particular substock.

### Animal age and productivity

We have implemented statuses for each substock that are determined by productivity (the ability to spawn), age, and additional information inserted by facility staff or researchers. Each status is visualized in a specific color to highlight the problematic substocks and to avoid mistakenly using them in an experiment. These basic statuses are unproductive, productive, retired, deceased, and sorted out. Moreover, temporary statuses can be turned on and off to visualize temporary productivity or progeny survival issues. The specific reason for the temporary statuses can be independently defined by each facility, based on their standard operating procedures.

### Breeding management

The crossing action serves for incrossing and outcrossing substocks in the database. When outcrossing substocks of different fishlines, a new fishline with the corresponding genotype combination is automatically created, unless it is already present in the database. Similarly to all other actions, crossing can be initialized both from the fish list and from the facility view. Moreover, Zebrabase enables users to distinguish automatically whether fish are hetero- or homozygous or whether zygosity is unknown for a specific genotype based on the zygosities of the parents. To plan or request crosses, the requesting functionality is accessible via the calendar tab.

### Genotyping

For the purposes of genotyping and sorting fish according to the transgene or mutation, two functionalities are available to improve the workflow. The action genotyping enables users to independently enter the zygosity of each genotype; if a more complex, physical rearrangement of the fish substocks is performed, a compound action split enables users to split the fish stock according to the genotypes, zygosities, or any other parameter, while they simultaneously enter the counts and positions.

### Workgroups and user rights

In case more research groups are sharing the facility and wish to have their data separated, the workgroup functionality enables to setup multiple workgroups with defined administrators, whose data will be completely separated in the user interface, although they are using a single database instance. However, the facility members (e.g., caretakers) can still retain the right to see the data of all workgroups sharing the facility, if required. Within each workgroup, several permission groups—administrator, facility staff, standard user, or guest—can be assigned to users ensuring their different rights. The permission groups have assigned permissions ranging from view-only to the most advanced administrator rights. In addition, a single user can be assigned to more permission groups, and the permissions automatically combine with each other.

### Calendar, requesting, and experiment planning

Submitting a request is a way to ask the facility staff for specific actions such as setting up fish for crossing, splitting, or terminating substocks and fishlines. Every request is then recorded in the database and shown in the calendar (Fig. 1C). E-mail notifications for new requests can be sent to the facility staff, if configured. The facility members are then able to accept the request, mark it as performed when the request is completed or decline it. Using the same functionality but assigning self-requests is a useful way to plan experiments. Self-requests are visible only to the user who created it. Generally, several request types can be entered: fish, setup fish, transfer substock, terminate substock, import, export, crossing, mobilize fish, and genotyping.

### Statistics and reporting counts and usage of the animals

Zebrabase uses a comprehensive reporting system of animal usage or death via the action count. Initially, the start count must be specified, which is typically performed when the juvenile fish are introduced into the facility system, or at any earlier or later time. Once this figure is available for a particular substock, the used or deceased fish can be subtracted, and the purpose of their euthanasia can be specified, if appropriate (e.g., type of an experiment). These records are then visualized in the facility overview (Fig. 1D) and can be used to generate reports.

### Data import and export

We provide a simple spreadsheet template that enables users to import a complete dataset, including the breeding history, to a new Zebrabase instance. Data exporting options include exporting statistics and full substock data (Supplementary Table S1; Supplementary Data are available online at www.liebertpub.com/zeb) into an.xls or .csv files and, upon request, complete database dumps can be provided.

## Methods

### System architecture

The Zebrabase system is operated with the utmost effort to ensure service availability. The system runs on a virtual server in an environment consisting of two clusters of hardware servers. Individual hardware components of the environment are redundant, and each cluster is housed in a different data center of the Institute of Molecular Genetics. The system is replicated from the primary to the secondary cluster every 30 min. In case the primary cluster becomes unavailable, the service can be restored in a short time on the secondary cluster by a system administrator. Due to rapid development of Zebrabase, a standalone version of the database where data would be stored at local servers is not available at the moment. However, we plan to provide such an option in the future under the condition of dedication to a single version of the database in the particular database instance.

### Database development

For every instance of Zebrabase, a dedicated PostgreSQL database (www.postgresql.org) is created to ensure high data security and consistency. The code base (Zebrabase version), both back- and front-end, is the same for all Zebrabase instances. For the back-end part of the application, the Python programming language (www.python.org) is used with the Django web framework (www.djangoproject.com), which facilitates data exchange between a web browser and the database. The front-end part is implemented using the latest HTML5/CSS3 technologies that enable the interface to be responsive and interactive and to use data visualizations. Zebrabase is a multiplatform (Windows, Mac OS, Android, iOS, and Linux-based systems) web application that works in all modern Internet browsers (Google Chrome, Mozilla Firefox, Microsoft Edge, and Apple Safari, among others).

### Data security and backup

All communication between users and the Zebrabase system is secured via Secure Sockets Layer (SSL), and a completely separate instance of Zebrabase is provided to each customer (e.g., facility or laboratory). Database dumps are performed four times a day and archived for 6 months when data restoration is necessary. In addition, fish stock records and statistics can be exported to an .xls or a .csv file directly from the user interface at any time to provide additional data backup.

### User interface

Zebrabase user interface provides full touch support and cross-platform access via web browser. Zebrabase implements an intuitive and consistent color coding, icons, and visualization in the Graphical User Interphase (GUI) that enables easy orientation in the fish substocks. User support is reachable via the feedback link at the bottom of every Zebrabase instance, which also provides an interface for suggestions of new features or improvements.

## Discussion

As the popularity of small fish species is increasing in research, the need for a proper tracking database that allows users to keep records of all animals, provides statistics and reporting capabilities, and enhances communication between users and caretakers at larger scale breeding facilities is critical. In this article, we propose a novel tracking database for aquatic model facilities, especially optimized for the use with small fish species such as zebrafish. Several major shortcomings of existing solutions have prompted us to develop Zebrabase, including commitment to a single platform, limited data export and transfer options, poor sustainability, and missing mobile device support. With Zebrabase, we tried to tackle these issues and to create a tracking solution that meets the needs of aquatic model facilities of any scale.

Zebrabase has been built around several key concepts that guarantee cross-platform operations, including full mobile device support and hosted maintenance-free service for users, unparalleled by any other solution. The cornerstones of Zebrabase are comprehensive animal tracking, interactive breeding history, and advanced management features. The animal tracking system of Zebrabase has been designed to carefully address the key specificities of the zebrafish tracking, primarily following a group of animals rather than tracking an individual animal, which substantially determines the data analysis and interpretation.

We have also focused on the reporting capabilities of the database, which allow users to store fish census and usage reports for the whole facility in a single excel file. This report provides a monthly overview of all fish born, deceased, euthanized, and used for experiments. We have also applied the suggestions of our beta testers and enhanced the requesting and experiment planning features of the database. The latest version now enables users to assign various types of requests either to the facility or to the users themselves so that all required actions in the fish facility are comprehensibly visualized. Advanced management features of Zebrabase, such as workgroups, user roles, responsible users, and QR code labeling will be a great benefit, especially for large facilities. Full touch and camera integration and QR code support are provided in the most recent Zebrabase version to ensure maximum system versatility and data accessibility from any device, which can be very beneficial for real-life use in fish facilities. In-App camera support is currently unavailable on Apple devices, but the substock records can be alternatively accessed via any mobile QR code reader application.

Zebrabase is a nonprofit project designed to support small and starting facilities by providing the service for free for up to 150 active substocks or 3 racks. After reaching this threshold, a yearly fee covering the initial setup, data backup and recovery, version updates, server maintenance, and user support is charged according to the size of the facility. For details on pricing, please visit the Zebrabase webpage (https://zebrabase.org/faq). To obtain the demo access or request a new database instance, please use the web form at https://zebrabase.org/contact.

Thanks to the hosted character of the Zebrabase project, we can provide unparalleled possibilities, including automatic backup and recovery options or instant version upgrades automatically available for all instances, regardless of whether they are running on the free or paid plan. The sustainability of this project is guaranteed because the tool is in use in the fish facility of the Institute of Molecular Genetics, where most of the new features are designed and thoroughly tested. Feedback from users is regularly collected and used to prioritize new features, and users are encouraged to report new suggestions for improvement to the Zebrabase team. In the near future, we plan to implement batch actions, enterprise resource planning and a genome editing module to streamline the workflow of researchers and facility managers, and to enable automated fee collection for the use of facility services. For more information about the new functionalities, please visit our webpage (https://zebrabase.org/news).

## Supplementary Material

Supplemental data
