# Supplementary material for: Zebrabase: An Intuitive Tracking Solution for Aquatic Model Organisms
Source: Zebrafish. 2018 Nov 29;15(6):642–7. doi: 10.1089/zeb.2018.1609 (PMC6277078; doi:10.1089/zeb.2018.1609)
Supplement: Supplemental data [file Supp_Table1.pdf]

ID	Name	Background	Num	DOB	Status	Count	Room	Rack	Position	Responsible	Use	Parent IDs
1003	b-actin:GFP+	AB	3/3	2016-06-21	retired	16	B	01	E04			
1295	ubi:mCherry+	TU	1/1	2016-06-22	retired	22	B	01	D05			741,741
1409	gli-d:mCherry+	None	3/3	2016-07-12	retired	22	B	01	C03	jana		
2781	mitfa:Hsa.BRAF_V600E+/-	None	1/1	2016-07-26	retired	3	B	01	E05	martina		827,827
1990	SHEER	None	1/1	2016-10-25	retired	10	B	01	D10			419,419
1366	ubi:GFP+/-	TU	1/2	2017-01-04	productive	20	B	01	D06			1232,996
1371	shh-ABC:GFP+/+	Unknown	2/3	2017-01-04	productive	20	B	01	C08	jana		773,773
1662	rag2:GFP+	AB	2/4	2017-01-06	productive	18	B	01	E06			1242,1242
1515	lyz:DsRed+	AB	1/2	2017-01-31	productive	23	B	01	C06			1353,1353
1632	cd41:GFP+	TU	1/2	2017-02-14	productive	20	B	01	E09	tereza		674,674
1506	mpeg1:GFP+/+	AB	1/2	2017-02-21	productive	20	B	01	B04	tereza		1014,1014
1496	7xTCF-xla.siam:NLS-mCherry+	Unknown	3/3	2017-02-23	productive	16	B	01	D03			872,872
1997	WT (AB)	AB	1/4	2017-02-23	productive	19	B	01	D09			627,627
1779	flk1:GFP+	None	1/1	2017-03-07	productive	20	B	01	B07	jana		989,989
1914	CASPER	AB	1/1	2017-03-07	productive	16	B	01	D04			1550,1550
1620	gata1:DsRed+/+	AB	1/1	2017-03-09	productive	20	B	01	B10			1517,1517
3254	c-myb:GFP-	AB	1/1	2017-03-21	productive	14	B	01	A02	jana		
2839	mhc2dab:GFP+	None	2/3	2017-03-28	productive	12	B	01	C07			1268,1268
2302	WT (AB) (mix)	AB	1/1	2017-04-19	productive	17	B	01	D07			1204,1204
2299	WT (TU)	TU	1/3	2017-04-27	productive	17	B	01	D08			2286,2286
3251	5xERE:GFP+/+	Unknown	2/2	2017-06-06	productive	9	B	01	B05			
3260	gfap:GFP+/+	Unknown	3/3	2017-06-21	productive	15	B	01	C02	jana		
2103	Xla.Tubb:DsRed+	Unknown	1/1	2017-06-21	productive	18	B	01	E10	jana		1405,1405
2079	-3.9nes:GFP+	Unknown	1/2	2017-06-28	productive	18	B	01	C10	jana		1856,1856
2084	mpx:GFP+	AB	1/1	2017-06-29	productive	17	B	01	B09			979,979
2090	flk1:NTR-mCherry+	Unknown	1/1	2017-06-29	productive	13	B	01	B08	jana		1734,1734
2262	drl:EGFP+/-	Unknown	1/3	2017-07-27	productive	18	B	01	B06	tereza		631,2161
3235	7xTCF-xla.siam:NLS-mCherry?	Unknown	1/1	2018-02-06	unproductive	20	B	01	A01			

Supplementary Table 1 – Substock Export File
Using the export function in the Fish tab, an .xls or .csv file can be created for a currently filtered set of fish, including basic substock information - ID, name, background, date of birth, status and parent information. Exported files can be used for reporting purposes or to provide an additional backup option.
